# Designing Microparticles of Luteolin and Naringenin in Different Carriers via Supercritical Antisolvent Process

**DOI:** 10.3390/polym16243600

**Published:** 2024-12-23

**Authors:** Stefania Mottola, Iolanda De Marco

**Affiliations:** 1 Department of Industrial Engineering, University of Salerno, Via Giovanni Paolo II, 132, 84084 Fisciano, Salerno, Italy; smottola@unisa.it; 2 Research Centre for Biomaterials BIONAM, University of Salerno, Via Giovanni Paolo II, 132, 84084 Fisciano, Salerno, Italy

**Keywords:** polyphenolic compounds, microparticles, supercritical carbon dioxide, carriers, controlled release

## Abstract

Antioxidants are contained in fruits and vegetables and are commonly obtained through food. However, it is frequently necessary to supplement the diet with substances that are often poorly soluble in water and sensitive to light and oxygen. For this reason, in this work, luteolin (LUT) and naringenin (NAR), two compounds with antioxidant activity and potential health benefits, were precipitated through the supercritical antisolvent technique using polyvinylpyrrolidone and β-cyclodextrin as the carriers. The precipitation occurred from dimethylsulfoxide using supercritical carbon dioxide as the antisolvent. The influence of pressure (9–12 MPa), active substance/carrier concentration in the solution (20–200 mg/mL), and their ratio (1/1 and 1/2 mol/mol) on morphology, particle mean size, and distribution were investigated. Under the optimized operating conditions, spherical microparticles with a mean diameter equal to 2.7 ± 0.9 μm (for LUT) and 5.5 ± 1.9 μm (for NAR) were obtained. The active ingredients were protected from the external environment by the presence of the carrier, and the dissolution rate was notably increased by processing them with β-cyclodextrin. It was sixty times faster and three times faster than that of the antioxidant alone for LUT and NAR, respectively.

## 1. Introduction

Flavonoids are polyphenols extensively present in fruit, vegetables, wine, and tea. More than 4000 different flavonoids have been identified and grouped into seven subclasses based on variations in the oxidation level of the central heterocycle: flavonols, flavones, isoflavones, anthocyanidins, flavanones, flavanols, and chalcones [1,2]. Flavonoids have diverse biological effects in many cell systems (in vitro and in vivo) [3,4]. Lately, significant attention has been directed toward their antioxidative characteristics and capacity to inhibit different phases of carcinogenesis [5]. Furthermore, some epidemiological studies have shown that the consumption of flavonoids leads to benefits in the case of cardiovascular diseases and tumors [6]. Soybeans, hypericum, milk thistle, ginkgo, various citrus, bilberries, hawthorn, tea, etc., are all medicinal plants containing flavonoids, and their efficacy in treating certain diseases has been proven by clinical studies [7].

Among the flavonoids, luteolin (LUT; C_15_H_10_O_6_) and naringenin (NAR; C_15_H_12_O_5_) are included in a wide selection of plants and fruits [8,9]. LUT belongs to flavones, the largest class of flavonoids, and is present in high concentrations in celery, parsley, thyme, green pepper, rosemary, and chamomile tea [10]. In recent years, it has been considered a potent natural drug, as its antimutagenic, antitumor, anti-inflammatory, and anti-allergic abilities have been proven [11,12,13]. NAR is commonly found in various citrus fruits and tomatoes and is the predominant flavanone in grapefruit [14]. It is a frequently used component in the human diet, garnering growing interest due to its beneficial impacts on health, particularly in cancer prevention [15] and in conditions beyond cancer, such as in the treatment of obesity [16] and as a myocardial infarction inhibitor [17].

The application of these active pharmaceutical ingredients (APIs) with relevant antioxidant activity and potential health benefits is hindered by their rapid degradation by light, temperature, the presence of oxygen, low solubility in aqueous systems, and low permeability within the intestine that result in small quantities of the molecules being available for oral administration. One way to overcome these disadvantages is to encapsulate the APIs in appropriate carriers, which can protect the molecules from external agents [18,19].

Innovative technologies using supercritical carbon dioxide (scCO_2_) have been developed to overcome some of the limitations of traditional technologies, such as (a) meager control of particle size and morphology, (b) decomposition of heat-sensitive compounds, (c) little encapsulation efficiency, (d) low precipitation yield, and (e) difficulty in completely removing solvents from the final product [20,21,22,23,24]. Indeed, using CO_2_ in the supercritical state allows processes to be carried out at temperatures close to room temperature, avoiding the decomposition of thermolabile compounds [25]. Different techniques have been implemented to produce microparticles and nanoparticles using supercritical CO_2_, both considering pure compounds and APIs encapsulated in appropriate carriers. These processes can be classified based on the role of scCO_2_ in the process (as a solvent, solute, or antisolvent) [26,27,28].

The most widespread of these processes is supercritical antisolvent precipitation (SAS), based on some prerequisites, such as the miscibility between an organic solvent and the scCO_2_ under the chosen operating conditions, the solubility of the API in the used solvent, and its insolubility in the scCO_2_-organic solvent mixture under process conditions [27,29]. If these prerequisites are respected, when the liquid solution containing the API is injected into a vessel containing scCO_2_, the latter mixes with the organic solvent and plays the role of an antisolvent toward the API, causing its precipitation in the form of particles. The SAS technique has been positively employed to precipitate microparticles and nanoparticles of pure APIs and to coprecipitate them with appropriate polymeric carriers [29]. It has been observed that some carriers, such as polyvinylpyrrolidone (PVP; (C_6_H_9_NO)n), allow the quickening of the dissolution of APIs (thus increasing their bioavailability) [30].

Promising results have also been obtained using β-cyclodextrin (β-CD, C_42_H_70_O_35_), an oligosaccharide that allows the formation of inclusion complexes due to the formation of weak bonds according to the so-called host/guest interaction. The cyclodextrin plays the role of the host thanks to its hydrophobic cavity into which the guest molecule enters [31,32,33].

Many papers have been published in the last ten years on the coprecipitation of APIs with PVP or cyclodextrins, with the aim of enhancing the dissolution rate. The active ingredients treated have the common characteristic of being poorly soluble in water, but they belong to the most disparate categories. For example, drugs such as aripiprazole, an antipsychotic drug [34], aprepitant, an antiemetic agent used to inhibit vomiting and nausea caused by cancer chemotherapy [35], and sulfadiazine, an antibiotic used to treat bacterial infections such as pneumonia, urinary tract infections, and meningitis [36] were successfully coprecipitated with PVP in the form of microparticles. Similarly, inclusion complexes with nimesulide and ketoprofen, widely used non-steroidal anti-inflammatory drugs [31], and tosufloxacin tosylate, a broad-spectrum antibacterial drug [37], were successfully prepared. Their scarce solubility in water and the necessity to protect them from oxygen and light is also a problem in the case of some flavonoids. For this reason, the SAS process has been employed to coprecipitate quercetin and rutin [38] with PVP, and apigenin [39] or hesperetin [40] with hydroxy-propyl-β-cyclodextrin.

To the best of our knowledge, luteolin and naringenin have never been coprecipitated with PVP or cyclodextrins via the SAS process. Therefore, to enhance the dissolution rate and, consequently, improve the bioavailability of LUT and NAR, the SAS technology was attempted under different operating conditions to produce composite microparticles containing the APIs using PVP and β-CD as the carriers.

## 2. Materials and Methods

### 2.1. Materials

The experiments were conducted using the following materials: carbon dioxide with a purity of 99%, purchased from Morlando Group S.p.A. (Sant’Antimo, Naples, Italy); dimethylsulfoxide (DMSO; purity: 99.5%), delivered by Carlo Erba (Cornaredo, Italy); luteolin (LUT), supplied by Epitech Group (Milan, Italy); and naringenin (NAR), β-cyclodextrin (β-CD; purity: 98%), and polyvinylpyrrolidone (PVP) with a molecular weight of 10 kg/mol, supplied by Sigma Aldrich (Milan, Italy).

### 2.2. SAS Plant and Experimental Procedure

**Figure 1:**
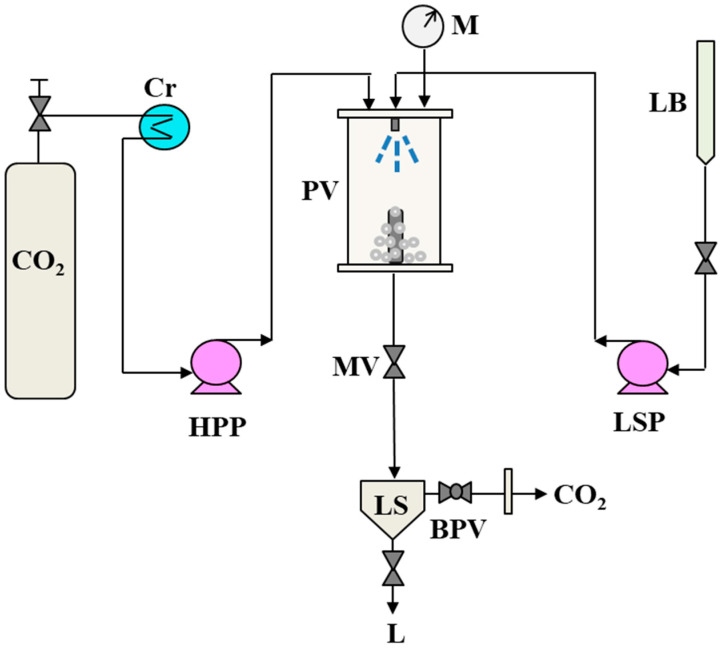
Schematic representation of the SAS apparatus. BPV: back-pressure valve; Cr: cryostat; HPP: high-pressure pump; L: liquid; LB: liquid burette; LS: liquid separator; LSP: liquid solution pump; M: manometer; MV: micrometric valve; PV: precipitation vessel.

A sketch of the SAS plant used for the experiment is reported in Figure 1.

The heart of the SAS plant was a stainless-steel cylindrical precipitation vessel (PV) with an internal volume of 500 cm^3^. CO_2_, which had the role of the antisolvent, was supplied to the vessel from a reservoir through a high-pressure pump (HPP). The liquid solution containing the solutes was charged in a burette (LB) and sprayed into the PV through a stainless-steel injector with the aid of another pump (LSP). Before being pumped, the CO_2_ was cooled through a cryostat (Cr) to avoid cavitation problems during pumping. A micrometric valve (MV) was mounted on the line downstream of the PV, which allowed the pressure inside the PV to be regulated. A PID controller was connected to two heating bands to maintain the desired temperature inside the vessel. In the lower part of the PV, a porous filter with a pore size of 0.1 μm allowed the solute to be retained inside the vessel and the CO_2_-organic solvent mixture to pass to a second vessel, which acted as a separator (LS). The pressure inside the LS was regulated with the aid of a back-pressure valve (BPV).

When the SAS test started, CO_2_ was sent into the PV until the experiment’s pressure and temperature were reached and stabilized. Then, the organic solvent was injected into the vessel. Once a quasi-stationary composition of the organic solvent and scCO_2_ was obtained, the liquid solution containing the solutes to be micronized was introduced into the PV through a 100 μm sized injector to promote the atomization of the liquid solution and the subsequent micronization of the solute. After the solution injection, CO_2_ continued to stream for 90 min to ensure the complete elimination of organic solvent residues. Once this time had elapsed, the CO_2_ pump was turned off, and the system was brought to atmospheric pressure.

In the tests carried out in this work, the chosen organic solvent was DMSO, and the temperature was fixed at 40 °C and the liquid flow rate at 1 mL min^−1^, whereas the CO_2_ flow rate was set to 30 g min^−1^, in accordance with previous results related to the production of microparticles consisting of a carrier and an active compound with the SAS process [29]. In each experiment, the test conditions, i.e., the pressure and concentration, were varied to comprehend the impact of the variation in these parameters on the results obtained. Each experiment was repeated twice. Before each test, solutions were prepared by dissolving the solute in 30 mL of DMSO (used as the organic solvent) to obtain the chosen concentration and the maximum sample yield. The scheme of the SAS plant has been depicted in a previous work [32].

### 2.3. Analyses

#### 2.3.1. Morphology of Powders and PSDs

A field-emission scanning electron microscope (FESEM, model LEO 1525, Carl Zeiss SMT AG, Oberkochen, Germany) was utilized to analyze the structure of the particles. Following each trial, the powder was carefully taken from various points of the filter and precipitation vessel, distributed onto carbon tabs, and coated with a layer of gold.

Sigma Scan Pro 5.0.0 from Aspire Software International was used to analyze the images and acquire the particle size distributions (PSDs). This software enabled the evaluation of the particle diameters. Additionally, the software Origin Pro 2021 from OriginLab Corporation (Northampton, MA, USA) was employed to calculate the size distributions and provide statistical data.

When the FESEM analysis revealed that the particles had nanometric dimensions, the PSDs were evaluated not only through image analysis but also using the Zetasizer instrument (model 5000) from Malvern Instruments Ltd. (Malvern, UK), utilizing dynamic light scattering (DLS). In this case, to prepare the suspension that had to be analyzed, the SAS-precipitated particles were placed in a glass cuvette containing ethyl acetate for CD-based inclusion complexes and acetone for PVP-based microspheres. A surfactant was added to stabilize the suspension created: Span80 for the β-CD-based powders and Tween 80 for the PVP-based powders. The liquid was then sonicated to guarantee a homogeneous suspension for the measurements to be carried out.

#### 2.3.2. Infrared Spectroscopy

The Fourier-transform infrared (FT-IR) spectra were analyzed utilizing an IRTracer-100 (Shimadzu Europa GmbH, Duisburg, Germany) at a resolution of 0.5 cm^−1^. Considering that the instrument measures the absorption from cylindrically shaped discs, each sample was prepared by blending 1 mg of powder with 100 mg of KBr powder. The investigation was then executed in a scan wavenumber range of 4000 to 500 cm^−1^.

#### 2.3.3. Dissolution Tests and Entrapment Efficiency

Dissolution tests were accomplished using a Cary 60 UV–vis spectrophotometer (Varian, Palo Alto, CA, USA). Either 5 mg of pure APIs or a specific amount of the SAS sample containing an equivalent quantity of APIs (5 mg) was suspended in 3 mL of PBS at a pH equal to 7.4 in the case of LUT [41] and 6.8 in the case of NAR [42] and placed in a dialysis sack. This sack was then immersed in 300 mL of PBS (to simulate physiological conditions), constantly stirred at 150 rpm, and preserved at 37 °C.

First, the calibration lines were determined using solutions of the active ingredients in a solution of PBS at different concentrations. In the case of LUT, the absorbance peak was obtained at a wavelength of 275 nm [41] and, in the case of NAR, at a wavelength of 288 nm [42]. Dissolution tests were then carried out for the pure APIs and for the API/carrier coprecipitated powder obtained through the SAS process. The tests were conducted until the plateau was reached, indicating that the active principle had completely migrated to the external phase. The analyses were repeated three times to ensure the results’ consistency and reliability.

The entrapment efficiency (EE, %) was evaluated as the ratio between the measured ($w_{M_{API}}$) and theoretical ($w_{T_{API}}$) active ingredient content (loaded in the precipitation vessel), using the ensuing formula:

(1) $$EE\%=\frac{w_{M_{API}}}{w_{T_{API}}}*100$$

#### 2.3.4. Job’s Method

The stoichiometry of the active compound/β-CD inclusion complexes was assessed using a continuous variation method, also known as Job’s working plot, named after the author who first used this method [43]. Two standard solutions were prepared, dissolving in distilled water the API and β-CD at equimolecular concentrations. Various volumes of these solutions were then mixed to obtain a constant total concentration (8 × 10^−5^ mmol/mL) with mole fraction X = [API]/([API] + [β-CD], ranging from 0 to 1. The resulting mixture was sonicated for 15 min and stirred at a constant temperature (25 °C) for 3 days to achieve equilibrium. After the solution was diluted, it was analyzed with a UV–visible spectrophotometer at a wavelength of 275 nm for LUT and 288 nm for NAR. The working curve was constructed by plotting ΔA × X vs. X, where ΔA represents the difference in API absorption in the absence and presence of β-CD.

The stoichiometry of the inclusion complex was determined by identifying the point on the graph corresponding to the maximum deviation of the physical property being monitored [44].

## 3. Results and Discussion

In this paper, the SAS process was used to precipitate inclusion complexes and coprecipitated particles containing two antioxidants, i.e., luteolin and naringenin. To obtain particles with an improved dissolution rate, the processing with two hydrophilic carriers, such as β-CD and PVP, was attempted, and the effects of some process parameters were studied. As reported in Table 1, the effects of pressure (P), the total concentration in the liquid solution (C_tot_), and the ratio between the API and the carrier on the morphology, mean diameter (m.d.), and standard deviation (s.d.) of the precipitated powders were studied.

### 3.1. Tests on Luteolin

A preliminary test (#1 in Table 1) was conducted by injecting a solution containing luteolin without any carrier, aiming to verify the API’s behavior following a SAS experiment. The test was carried out in correspondence with standard operating conditions in the case of the SAS process, such as a temperature of 40 °C, a pressure of 9 MPa, and a concentration of LUT in DMSO equal to 20 mg/mL. At the opening of the precipitation chamber, a small quantity of powder was observed, demonstrating that a high amount of luteolin remained dissolved in the DMSO-scCO_2_ mixture and was entrained in the separator. This result can be explained because, evidently, under the chosen process conditions, luteolin had a non-negligible solubility in the scCO_2_-organic solvent mixture and, therefore, scCO_2_ was unable to carry out its antisolvent effect. This preliminary test confirmed that it is necessary to use a carrier to precipitate LUT in the powder form.

#### 3.1.1. Tests with β-Cyclodextrin

**Figure 2:**
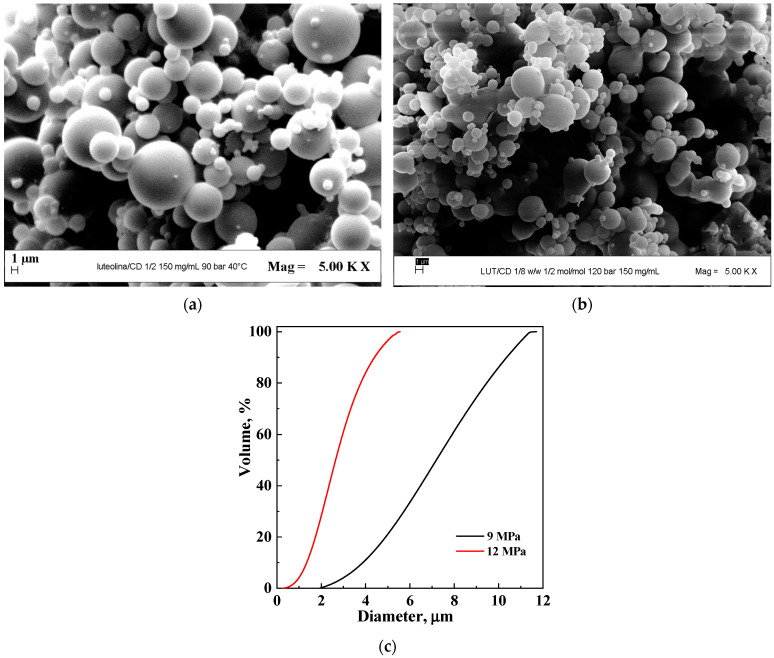
Luteolin/β-CD powder precipitated at 150 mg/mL and 1/2 LUT/β-CD molar ratio. The effect of pressure: (**a**) FESEM image at 9 MPa; (**b**) FESEM image at 12 MPa; (**c**) comparison of PSDs.

The initial series of coprecipitation experiments utilized β-CD as the carrier. First, the impact of the total concentration of the liquid solution was evaluated, with the API/carrier ratio fixed at 1/2 mol/mol and the pressure set at 9 MPa. When the concentration was maintained at 150 mg/mL (#2 in Table 1), well-defined microspheres were attained, as shown in Figure 2a. In contrast, when the concentration was raised to 200 mg/mL (#3 in Table 1), only a liquid phase was recuperated at the end of the test, indicating that this concentration was too high.

To estimate the effect of the pressure on the morphology and the mean diameter of the powders, a test increasing the pressure to 12 MPa was carried out (#4 in Table 1), maintaining the total concentration at 150 mg/mL and the molar ratio at 1/2. Also, in this case, micrometer-sized particles were obtained; the corresponding FESEM image is reported in Figure 2b. Upon comparing the cumulative volumetric distributions (Figure 2c), it becomes evident that the elevation in pressure resulted in a decrease in the average diameter and a reduction in the amplitude of the PSD. This result is in agreement with the results obtained previously in the literature [45].

**Figure 3:**
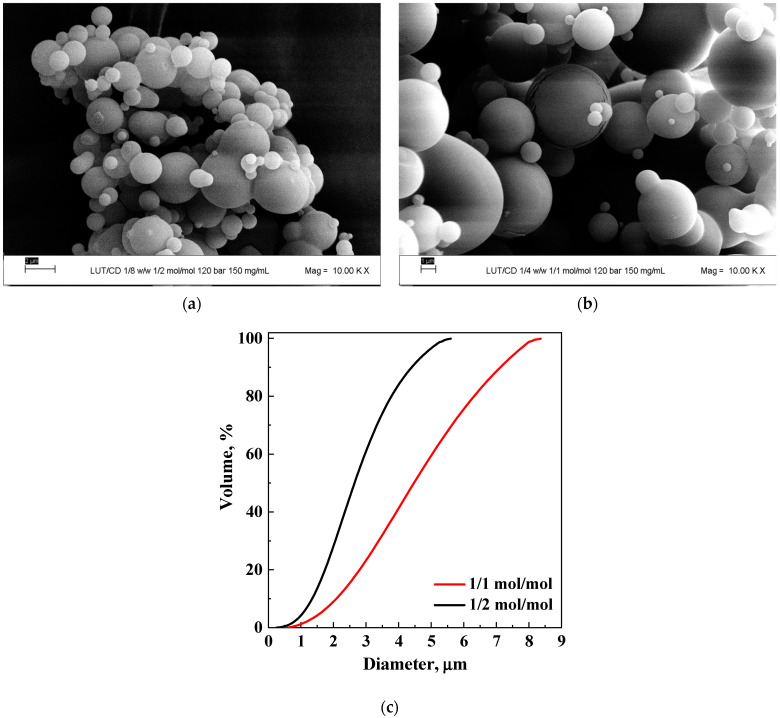
Effect of luteolin/β-CD molar ratio on the powders precipitated at 150 mg/mL and 12 MPa: (**a**) FESEM image at 1/2 mol/mol; (**b**) FESEM image at 1/1 mol/mol; (**c**) comparison of the PSDs obtained at equimolar ratio (X equal to 0.5) and 1/2 molar ratio (X equal to 0.33).

In the next set of experiments, the effect of the LUT/β-CD molar ratio was studied, setting the concentration at 150 mg/mL and the operating pressure at 12 MPa. In particular, the results obtained by fixing the molar ratio at 1/2 and 1/1 were analyzed. Regular microparticles were obtained in correspondence with both the molar ratios, as it is possible to observe in the FESEM images reported in Figure 3a,b. The comparison of the corresponding PSDs in terms of the volumes of the particles is shown in Figure 3c.

The mean diameter increased by varying the LUT/β-CD molar ratio from 1/2 to 1/1. This result seems “anomalous” compared with those obtained in the previous literature regarding luteolin processed through supercritical-based processes (different from SAS), in which the average size of the particles generally increases with increasing quantity of the carrier, i.e., as the drug/carrier molar ratio decreases [41,46]. However, the trend obtained in the present work, which detected an increase in the average diameter of the particles as the active ingredient/carrier ratio increased, agrees with some of the literature regarding the formation of CD-based complexes using the SAS technique [31].

Indeed, the result can be justified by considering the thermodynamic aspects linked to the equilibria involved in the SAS process.

It is known that the presence of solutes can influence the phase equilibria of the binary DMSO-CO_2_ system [47]. By increasing the amount of active compound, it was possible that there was a shift in the mixture critical point (MCP) of the β-CD/active compound/DMSO/CO_2_ quaternary system toward higher pressures than those of the MCP of the binary system consisting only of the solvent and antisolvent. This would have caused the operating point of the luteolin/β-CD ratio of 1/1 mol/mol to be closer to the MCP, resulting in larger particles than when tested at a 1/2 molar ratio, thus producing smaller particles.

#### 3.1.2. Tests with PVP

**Figure 4:**
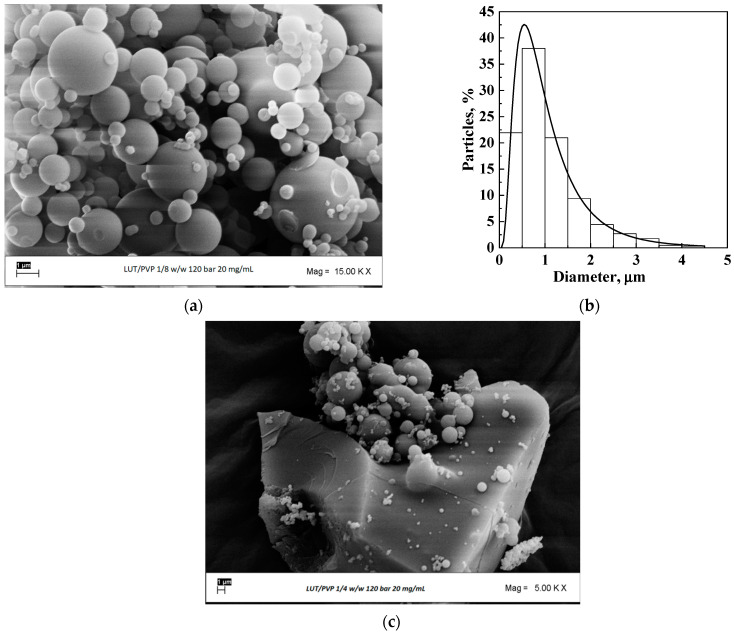
Luteolin/PVP powder precipitated at 20 mg/mL and 12 MPa; (**a**) FESEM image at 1/2 mol/mol; (**b**) particle size distribution at 1/2 mol/mol; (**c**) FESEM image at 1/1 mol/mol.

Once the best operating conditions that allowed obtaining micrometric particles using β-CD as the carrier were identified, some tests using PVP were performed. The concentration of the solutes in the liquid solution was fixed at 20 mg/mL because the solubility of LUT + PVP in DMSO was lower than that of LUT+ β-CD in the same solvent. The pressure was imposed at 12 MPa, whereas the ratio between LUT and PVP was fixed at 1/2 mol/mol and 1/1 mol/mol. In the first case, well-defined microparticles with a mean diameter equal to 2.2 μm were precipitated, as it is possible to observe in the FESEM image reported in Figure 4a and from the particle size distribution reported in Figure 4b. Increasing the LUT/PVP molar ratio to 1/1 mol/mol, crystals with few microparticles on them were obtained, as reported in Figure 4c; therefore, the coprecipitation failed when the amount of luteolin with respect to PVP was increased.

Comparing the results obtained with the two carriers, it is evident that the use of β-CD allowed the possibility of obtaining microparticles from a broader range of operating conditions, whereas, using PVP, it was possible to precipitate spherical particles only with a lower amount of the API. In any case, the analyses were conducted on the optimized powders containing luteolin obtained with both carriers.

### 3.2. Tests on Naringenin

Similarly to what was performed previously with luteolin, a feasibility test (#8 in Table 1) in the absence of a carrier was carried out by processing naringenin alone. As observed in the case of luteolin, a small quantity of powder was recovered when the test was concluded, as the naringenin was almost wholly extracted from the DMSO/scCO_2_ mixture. Therefore, in this case, it was verified in the subsequent experiments whether β-cyclodextrin and PVP allowed the coprecipitation of composite microspheres.

#### 3.2.1. Tests with β-Cyclodextrin

When investigating the naringenin/β-cyclodextrin system, the consequence of the pressure and the API/carrier ratio variation on the morphology, size, and particle size distribution of the powders was assessed.

**Figure 5:**
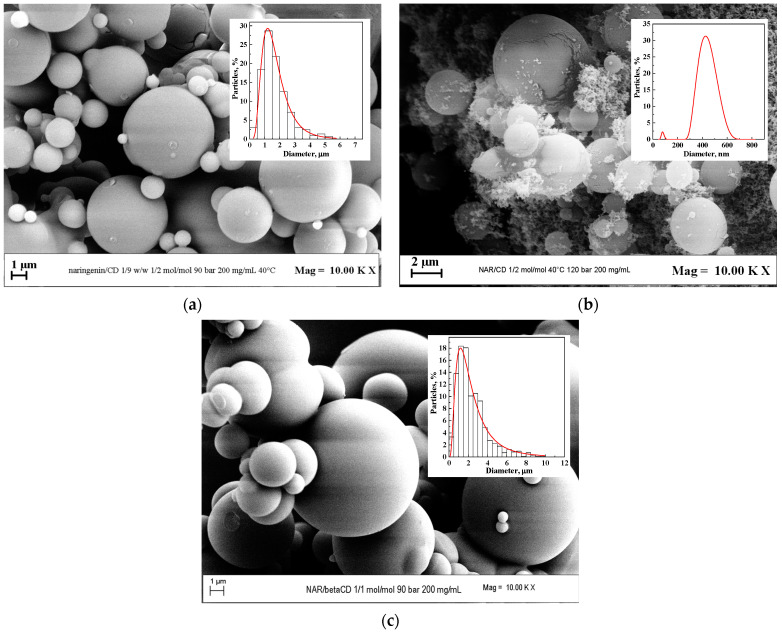
Naringenin/β-CD powder precipitated at 200 mg/mL and 40 °C; (**a**) FESEM image and PSD at 9 MPa and 1/2 mol/mol; (**b**) FESEM image and PSD at 12 MPa and 1/2 mol/mol; (**c**) FESEM image and PSD at 9 MPa and 1/1 mol/mol.

To evaluate the pressure effect, the temperature was held constant at 40 °C, the active substance/carrier ratio was maintained at 1/2 mol/mol, and the total concentration was set at 200 mg/mL. The experiments were carried out at 9 and 12 MPa. In correspondence with the lower pressure, spherical well-separated particles with a mean diameter of 2.9 μm were obtained, as it is possible to observe from the FESEM image and the corresponding PSD reported in Figure 5a. Increasing the pressure to 12 MPa, a non-homogeneous morphology was obtained since the samples analyzed under FESEM showed the presence of both microparticles and nanoparticles, as evident from the image reported in Figure 5b. So, to avoid the attainment of an unreliable PSD through the image analysis software, the Zetasizer tool was used to calculate the particle size distribution, which curve is shown in the upper right corner of Figure 4b. The particle size curve obtained was bimodal, which confirmed the irregular morphology of the sample, visible in the FESEM image. The average size was about 78 nm for the first nanoparticle-bound peak and 425 nm for the second peak.

Once the value of the operating pressure was optimized, the possibility of increasing the active ingredient/carrier ratio to 1/1 mol/mol was investigated. Again, spherical microparticles with a mean diameter of 5.5 μm (see the FESEM image and the PSD reported in Figure 5c) were obtained.

Comparing the distributions obtained at 1/2 mol/mol and 1/1 mol/mol, it is possible to note an increase in the mean diameter with the rise in the antioxidant amount. This is the same as the experimental evidence previously observed in the case of luteolin. This trend can be traced back to the effect that the presence of a higher quantity of naringenin had on the phase equilibria of the quaternary β-CD/naringenin/DMSO/CO_2_ system, for which the MCP was shifted toward higher pressure values than that of the solvent/antisolvent binary system. As a result, by decreasing the naringenin/β-CD ratio, i.e., by reducing the amount of naringenin, the operating point moved away from the MCP, resulting in smaller-diameter particles.

#### 3.2.2. Tests with PVP

**Figure 6:**
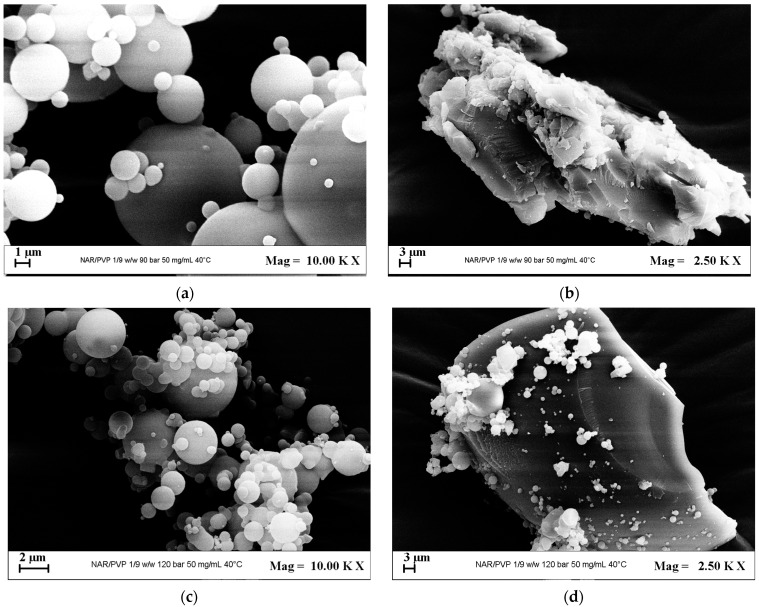
FESEM images of naringenin/PVP powders precipitated at 50 mg/mL, 1/2 mol/mol, and 40 °C; (**a**) microparticles obtained at 9 MPa; (**b**) crystals obtained at 9 MPa; (**c**) microparticles obtained at 12 MPa; (**d**) crystals obtained at 12 MPa.

When PVP was used as the carrier, the effect of the operating pressure on the morphology and size of the micronized naringenin powders was assessed. As in the case of luteolin, with NAR, the maximum solubility of API/PVP in DMSO was lower than that of API/β-CD. Indeed, it was possible to perform the experiments with NAR/PVP at a maximum concentration equal to 50 mg/mL. The other operating parameters were a temperature of 40 °C and an API/PVP ratio equal to 1/2 mol/mol. Well-defined particles and crystals were obtained using a pressure of 9 MPa and a pressure of 12 MPa. The FESEM images related to the test at 9 MPa are reported in Figure 6a,b, whereas the FESEM images related to the test at 12 MPa are reported in Figure 6c,d.

### 3.3. Characterization

FT-IR analyses were followed up to prove the occurrence of relations between the functional groups of active ingredients and the carriers. In Figure 6, the spectra of the pure active compounds, β-CD, PVP, physical mixtures, and coprecipitated powders at different API/carrier ratios are reported and compared.

**Figure 7:**
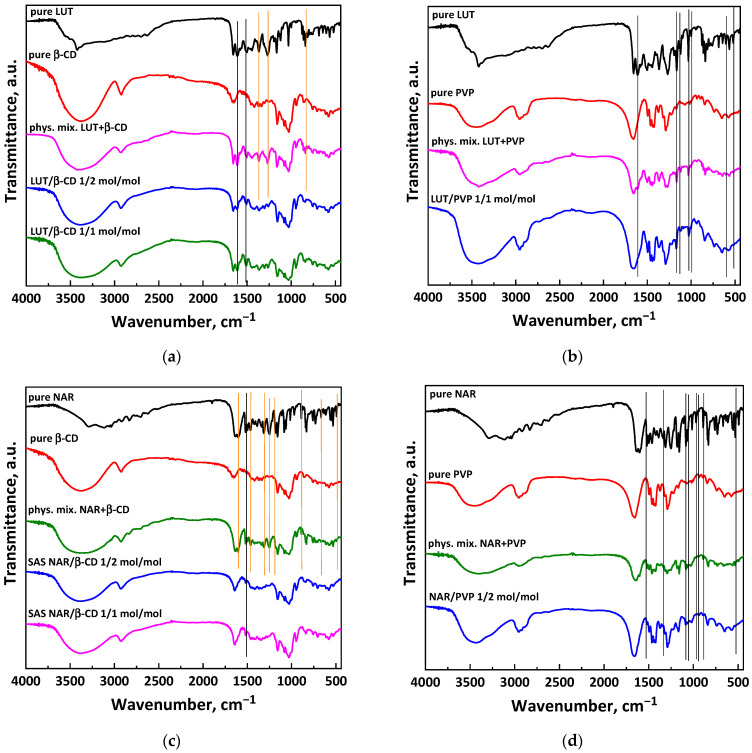
FT-IR spectra of the pure compounds, physical mixtures, and coprecipitated powders: (**a**) LUT/β-CD; (**b**) LUT/PVP; (**c**) NAR/β-CD; (**d**) NAR/PVP.

The spectrum of β-CD (red curve in Figure 7a,c) reveals a wide absorption band at 3371 cm^−1^, which belongs to the stretching vibrations of the OH group; a band at about 2928 cm^−1^, corresponding to the stretching of the CH_2_ bond; and three bands with frequencies of 1022, 1633, and 1153 cm^−1^ due to the C=O stretching of the glycosidic bond, of the cyclic alcohol, and of the primary alcohol, respectively [48]. As for the spectrum related to pure PVP (red curve in Figure 7b,d), this presents a wide absorption band at 3434 cm^−1^, which indicates the -OH group vibrational stretching; a peak at 2955 cm^−1^, an index of the vibrational stretching of the C-H bond; a peak at 1653 cm^−1^, indicating the vibrational stretching of the C=O double bond; two absorption bands relative to the CN group for wavenumbers equal to 1496 and 1463 cm^−1^; and two absorption bands at 1440 and 1291 cm^−1^, revealing the presence of CH groups [45].

Considering the two APIs (black curves in Figure 7), it is possible to observe that pure luteolin has a peak at 1654 cm^−1^, related to the vibrational stretching of the C=O double bond; an absorption band detected at 1610 cm^−1^, due to the vibrational stretching of the C=C double bond; and two absorption bands, referring to the vibrational stretching of the C-OH and C-O-C bonds, at 1262 cm^−1^ and 1158 cm^−1^, respectively [49]. Regarding the spectrum for pure naringenin, this reveals the presence of peaks at 1510 and 1601 cm^−1^, which can be attributed to aromatic bonds; two bands at 1200 and 1316 cm^−1^ due to the presence of OH bonds characteristic of phenolic compounds; and an absorption band detected at 1658 cm^−1^ due to the vibrational stretching of the C=O double bond [50,51,52].

The SAS luteolin/β-CD spectra (Figure 7a) are similar to that of pure β-CD, except for the presence of a couple of bands characteristic of pure luteolin (black vertical lines). Meanwhile, the characteristic bands of the latter are more present in the physical mixture (black + orange vertical lines). These results indicate that the complexation between the antioxidant and β-CD nevertheless occurred. Moreover, luteolin seemed to be more incorporated into the CD cavity even at higher antioxidant/CD ratios, i.e., in the presence of more luteolin, a further symptom of the formation of inclusion complexes. In the case of the LUT/PVP system (Figure 7b), the spectra of the physical mixture and the SAS processed sample are a combination of the spectra of the polymer and the luteolin as is. This indicates the presence of both PVP and luteolin in the coprecipitates, although the polymer bands dominate the spectra of both the physical mixture and coprecipitates being present in high quantities.

As can be seen from Figure 7c, while the NAR/β-CD physical mixture has different peaks characteristic of pure NAR, the spectra of the NAR/β-CD coprecipitated powders both at 1/2 and 1/1 mol/mol detect only the peaks that are characteristic of the carrier, except for the peak at 1510 cm^−1^. This indicates the formation of the guest/host inclusion complex, particularly a total complexation in the NAR/β-CD case.

For the naringenin/PVP system (Figure 7d), the spectra of the physical mixture and the SAS-processed sample are a combination of the polymer spectra and the pure naringenin.

**Figure 8:**
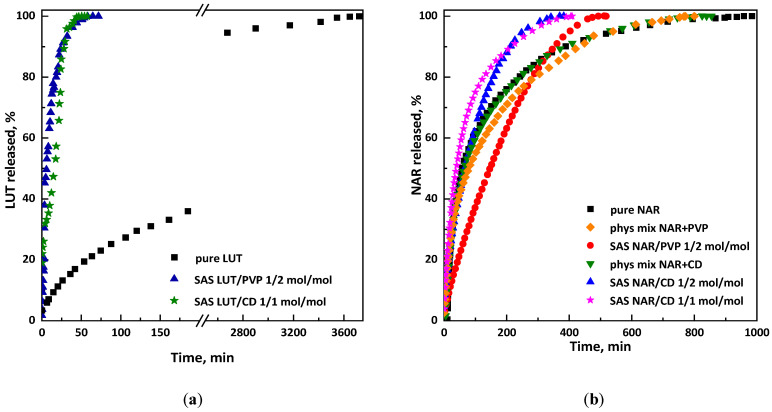
Comparison of release kinetics: (**a**) luteolin and (**b**) naringenin.

Release tests were carried out to verify the effect of coprecipitation on the dissolution rate. Regarding the LUT/β-CD system, it is possible to compare the release profiles of untreated luteolin, LUT contained in the inclusion complex precipitated at a 1/1 mol/mol ratio, and LUT contained in the sample produced at a 1/2 mol/mol luteolin/PVP ratio (since it is the only test that led to the production of well-defined composite microspheres and, thus, to effective coprecipitation). Figure 8a clearly shows the remarkable difference between the curve representing the unprocessed luteolin, which reaches complete dissolution in PBS in about 60 h, with respect to the curve indicating the dissolution profile of the coprecipitated powder, as a result of the SAS process, at a luteolin/β-CD ratio of 1/1 mol/mol, which took just over 50 min to reach a plateau.

Similar results were obtained in the case of the luteolin/PVP system. As can be seen from Figure 8a, the curve representing this sample reaches complete dissolution in solution in about 70 min. Comparing the release kinetics for both luteolin/carrier systems, it is observed that the dissolution times are practically identical; however, it should be noted that when the CD was used, a lower amount of carrier was used compared with PVP, with the same morphology of the particles. Therefore, the CD represents a better solution from a pharmaceutical point of view.

The dissolution tests of naringenin are shown in Figure 8b; it can be observed that pure naringenin took about 17 h to dissolve entirely in PBS, the physical mixtures NAR/PVP and NAR/β-CDs had almost the same curve as the pure antioxidant, and both arrived at complete dissolution in about 13 h, while the NAR/β-CD coprecipitates dissolved entirely in about 5 h. The NAR/PVP co-precipitates underwent almost a slowdown in release. This is because, with the NAR/PVP system, there was probably no effective coprecipitation, in accordance with what was found in the FESEM images showing microparticles and the presence of crystals. Therefore, it is possible to say that the carrier that allows a better bioavailability of the selected active compounds is β-cyclodextrin.

**Figure 9:**
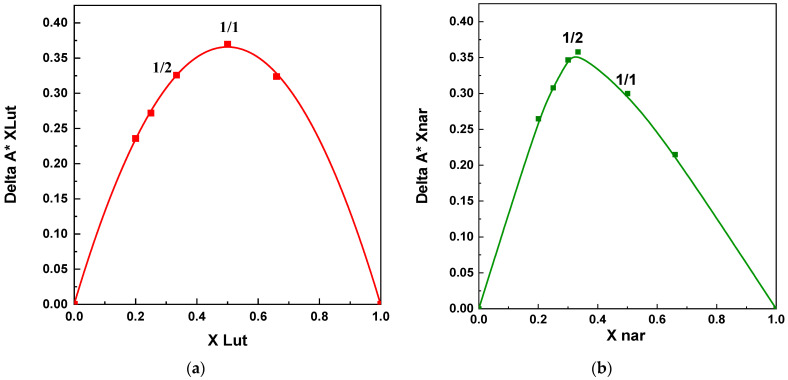
Job’s plots of the inclusion complexes: (**a**) luteolin and (**b**) naringenin.

To assess between the two tested ratios, i.e., which of them corresponded to the optimal stoichiometric guest-to-host molecule ratio for the formation of stable inclusion complexes between the APIs and β-CD, Job’s plots were evaluated (see Figure 9). It is possible to observe that the maximum values occurred at different values for luteolin and naringenin, with X equal to 0.5 (corresponding to an equimolar ratio in the case of luteolin) and X equal to 0.33 (corresponding to a 1/2 molar ratio) for naringenin.

## 4. Conclusions

This is the first study related to the application of the SAS technique for the coprecipitation of luteolin and naringenin with two different carriers. It was demonstrated that the use of inclusion complexes with β-CD or coprecipitated powders with PVP can accelerate the dissolution of the studied active ingredients and, therefore, allows the achievement of the set objectives; in particular, the dissolution rate of LUT is sixty times faster by using both β-CD and PVP as the carrier, whereas in the case of NAR, the dissolution rate is three times faster using β-CD as the carrier. The obtaining of micrometric particles of the desired range and of a regular size allows the active compounds to be appropriately conveyed. For both the active principles, the use of β-CD seems more advantageous than PVP, as it allows us to diminish the carrier’s amount in the composite powder, ensuring a rapid release and allowing the active ingredient to dissolve faster, thus improving its bioavailability. It will be important to review the dosage of the active ingredient to be loaded and prepare powders containing several suitably matched nutraceutical active ingredients.

## Figures and Tables

**Table 1:** SAS experiments carried out at 40 °C to produce PVP-based coprecipitated particles and β-CD-based inclusion complexes (EE = entrapment efficiency, MP = microparticles, NP = nanoparticles, Liq = liquid, and C = crystals).

#	API	Carrier	API/Carrier (mol/mol)	P (MPa)	Ctot (mg/mL)	Morphology	m.d. ± s.d. (μm)	EE%	Figure
1	LUT	-	-	9	20	-	-	-	
2		β-CD	1/2	9	150	MP	7.2 ± 2.3	100	2a
3				9	200	Liq	-	-	
4				12	150	MP	2.7 ± 0.9	100	2b,3a
5			1/1	12	150	MP	4.5 ± 1.4	100	3b
6		PVP	1/2	12	20	MP	2.2 ± 0.7	100	4a
7			1/1	12	20	C	-	-	4c
8	NAR	-	-	9	20	-	-	-	
9		β-CD	1/2	9	200	MP	2.9 ± 0.9	100	5a
10				12	200	MP + NP	-	-	5b
11			1/1	9	200	MP	5.5 ± 1.9	100	5c
12		PVP	1/2	9	50	MP + C	3.0 ± 0.9	-	6a,6b
13				12	50	MP + C	1.3 ± 0.5	-	6c,6d

## Data Availability

The data will be made available upon request.
